# Inflammatory markers and incident heart failure in older men: the role of NT-proBNP

**DOI:** 10.2217/bmm-2020-0669

**Published:** 2021-03-12

**Authors:** Douglas GJ McKechnie, A Olia Papacosta, Lucy T Lennon, Paul Welsh, Peter H Whincup, S Goya Wannamethee

**Affiliations:** 1 Department of Primary Care & Population Health, University College London, London, UK; 2 Institute of Cardiovascular & Medical Sciences, University of Glasgow, Glasgow, UK; 3 Population Health Research Institute, St George's University of London, London, UK

**Keywords:** B-type natriuretic peptide, biomarkers, cardiovascular disease, cohort studies, heart failure, inflammation

## Abstract

**Aim:**

To determine the relationship between baseline inflammation (CRP and IL-6) with natriuretic peptide (NP) activity (measured by NT-proBNP) and incident heart failure (HF) in older men.

**Methods & results:**

In the British Regional Heart Study, 3569 men without prevalent myocardial infarction or HF were followed for mean 16.3 years; 327 developed HF. Baseline CRP and IL-6 were significantly and positively associated with NT-proBNP. Those in the highest CRP and IL-6 quartiles had an elevated risk of HF after age and BMI adjustment (HR = 1.42 [1.01–1.98] and 1.71 [1.24–2.37], respectively), which markedly attenuated after NT-proBNP adjustment (HR = 1.15 [0.81–1.63] and 1.25 [0.89–1.75], respectively).

**Conclusion:**

NP activity is associated with pro-inflammatory biomarkers and may explain the link between inflammation and incident HF.

## Lay abstract

Inflammation describes the body’s natural response to infections, injuries and toxins. Inflammation is a helpful response in the short term, but it is thought that long-lasting inflammation – for example, due to illnesses such as diabetes or obesity – may have harmful effects. Previous studies have found that people with higher levels of inflammatory molecules in the blood seem to be more likely to develop heart failure (HF) later on. The amount of fluid in the body is controlled, in part, by molecules in the blood known as ‘natriuretic peptides' (NPs). People with HF have much higher levels of NPs in their blood, and these are used to help diagnose HF. There are suggestions that inflammation and natriuretic peptides are linked to one another. Using a sample of men aged 60–79 years, who did not have HF, we compared blood markers of inflammation and NPs at a baseline examination. Men with higher blood inflammatory markers tended to have higher blood NP levels. We then followed these men up for an average of 16.3 years. Men with higher blood inflammatory markers at baseline were more likely to develop HF, as expected, even after accounting for differences in age and BMI. However, when we accounted for NP levels at baseline, the increased risk of HF with inflammation disappeared. This suggests that NP activity is important in the relationship between inflammation and the risk of HF. Future studies should account for this when examining the link. It is possible that NPs or, more likely, whatever is driving their release, may explain why people with inflammation are more likely to get HF.

Heart failure (HF) is a major cause of morbidity and mortality globally, and its prevalence is predicted to increase with aging population demographics [1]. Chronic activation of the immune system is generally thought to be central to the development and progression of HF and its subtypes, HF with reduced ejection fraction (HFrEF) and HF with preserved ejection fraction (HFpEF) [2].

Circulating biochemical markers of inflammation, when elevated – even in the absence of clinical cardiovascular disease – have been shown to be associated with an increased risk of incident HF, persisting despite adjustment for ‘traditional’ cardiovascular risk factors: these include C-reactive protein (CRP) [3–14]; IL-6 [5,8,10,11,15]; TNF-α [8,10]; erythrocyte sedimentation ratio [16]; total blood white cell count [12]; blood granulocyte count [17]; growth differentiation factor-15 [18] and soluble ST2 (sST2) [18]. IL-6 is considered an upstream inflammatory cytokine which is a central mediator of the acute-phase response, and is essential to the initiation and progression of atherosclerosis [19]. Upstream IL-6 leads to the hepatic production of the downstream acute-phase reactant CRP. Experimental studies have suggested that pro-inflammatory cytokines (e.g., IL-6, TNF-α) may play a role in stimulating cardiac fibrosis and left ventricular remodeling [20,21]. Natriuretic peptides (NPs), such as B-type natriuretic peptide (BNP) and amino–terminal fragment of pro-B-type natriuretic peptide (NT-proBNP), markers of left ventricular stress, are used to aid diagnosis of acute and chronic HF in symptomatic patients [22]. BNP and NT-proBNP are produced by cleavage of the prohormone proBNP. NT-proBNP itself is biologically inactive, but is more stable at room temperature and subject to less intra- and inter-individual variation than BNP, meaning its use is generally favored as a proxy measurement of NP production and thus NP system activation [23]. Elevated levels of NT-proBNP in people without baseline cardiovascular disease strongly predict the onset of subsequent HF [24,25].

Data on the relative association between inflammatory markers and HF risk are conflicting. In the ARIC study, adding CRP to risk scores incorporating NT-proBNP did not improve incremental risk prediction [26]. In the British Regional Heart Study, we have previously shown that NT-proBNP improved HF prediction beyond that offered by use of traditional risk factors, but CRP did not [25]. In contrast, analysis of the PROGRESS study reported that NT-proBNP and CRP were both independent predictors of HF risk in patients with stroke [27]. A recent cohort study in middle-aged participants showed that CRP predicted incident HF independent of NT-proBNP, while IL-6 did not predict HF [6]. In contrast, two studies in older adults found that IL-6, but not CRP, predicted HF [8,10]; neither of these assessed the influence of NT-proBNP. This difference in findings might relate to a difference in prevalence of HFrEF and HFpEF in different age groups. HFpEF tends to be more common in older adults [28] and CRP has been reported to be less strongly associated with HFpEF compared with HFrEF [29], although conflicting results report that inflammation is predominant in HFpEF and not HFrEF [30].

Using a large cohort of older men, we aimed to determine the relationship between a pro-inflammatory cytokine (IL-6), an acute phase reactant (CRP) and incident HF (including incident HFpEF and HFrEF, considered together and then separately) over a long follow-up period. We also aimed to assess the additional role of NT-proBNP in defining this relationship, including whether or not the well-established relationship between NT-proBNP and incident HF attenuates any effect of inflammatory activation on HF risk, which might suggest interlinkage between the neurohormonal and inflammatory systems and the development of HF.

## Materials & methods

### British Regional Heart Study

The British Regional Heart Study was a prospective study of 7735 men, aged 40–59 years at enrollment, drawn from one general practice in each of 24 British towns. The sample was chosen to reflect the socioeconomic makeup of those towns, and was predominantly of White European ethnicity (>99%) [31]. Ethical approval was obtained from all relevant local Research Ethics Committees, and all subjects provided their informed consent to participate.

Initial screening took place from 1978 to 1980, and surviving participants were invited to follow-up examinations after 20, 30 and 40 years. They were followed up for mortality and cardiovascular morbidity throughout this period. The present report is based on the 20-year examination upon which the analyses described here are based, henceforth referred to as the ‘baseline’ for this paper, and associated follow-up.

All participants who took part in the baseline examination completed a questionnaire regarding their lifestyle and medical history, had a physical examination, and provided a fasting blood sample. Twelve-lead ECGs were recorded using a Siemens Sicard 460 instrument and classified using the Minnesota Coding scheme [32]. Prevalent HF was defined as a physician diagnosis of HF prior to baseline (based on review of primary care records) or self-report of a diagnosis of HF.

All men were followed up to June 2016 for cardiovascular morbidity and mortality through general practitioners’ medical records and the National Health Service Register for mortality. Follow-up has been achieved for 99% of the cohort [33]. Evidence of nonfatal HF or myocardial infarction (MI) was obtained by *ad hoc* reports from the participants’ general practitioners, supplemented by biennial reviews of their medical records (including hospital and clinical correspondence). All cases were verified by a review of available clinical information from primary and secondary records (including symptoms, signs, investigations and treatment response). Incident fatal HF was defined as HF that was mentioned as the underlying cause of death on death certificates (ICD-9 code 428). Incident HF included both incident non-fatal and incident fatal HF.

General practitioners were asked if participants with HF had an echocardiogram performed, and, furthermore, if it showed a diminished left ventricular ejection fraction (LVEF). Participants with incident HF were classified into ‘probable HFrEF’ if the LVEF was reported as reduced, ‘probable HFpEF’ if a normal LVEF was reported or ‘unknown’ if no information regarding LVEF was given.

### Biomarker measurement

Estimated glomerular filtration rate (eGFR) was calculated from serum creatinine measurements using the Modification of Diet in Renal Disease equation [34]. NT-proBNP was determined using the Elecsys 2010 electrochemiluminescence method (Roche Diagnostics, Burgess Hill, UK), as described previously [25]. High-sensitivity CRP was assayed by ultra-sensitive nephelometry (Dade Behring, Milton Keynes, UK). IL-6 was assayed using a high-sensitivity ELISA (R & D Systems, Oxford, UK).

### Exclusion criteria

Men with a diagnosis of HF at baseline were excluded, as were men with a prior diagnosis of MI. Men with ischemic heart disease are at considerably higher risk of incident HF and tend to have elevated levels of inflammatory markers [35] and NPs [36]; we therefore aimed to avoid confounding from complications of ischemic heart disease.

### Statistical analysis

All statistical analyses were performed using SAS software, version 9.4 of the SAS System for Windows (NC, USA). Statistical significance was set at p-value <0.05.

Descriptive statistics were used to report sample characteristics at baseline. Comparisons of these characteristics between the HF outcome groups were performed with the chi-square test for categorical variables; the *t* test was used for normally distributed continuous variables (BMI, high-density lipoprotein [HDL] and blood pressure). The distribution of NT-proBNP, CRP and IL-6 were positively skewed, and so geometric means were calculated for these variables and comparisons at baseline made using the Kruskal–Wallis test. These variables were natural log-transformed for use in regression analyses.

Multiple regression analyses were performed to determine the association between CRP and IL-6 quartile (as independent variables) with log NT-proBNP (as a dependent variable), initially crudely, then adjusted for age, and further adjusted for age, BMI, systolic blood pressure, HDL, eGFR (all modeled as continuous variables), CRP or IL-6 quartile (1/2/3/4), social class (manual vs nonmanual), smoking status (never smoked/stopped smoking ≥15 years before baseline/stopped smoking <15 years before baseline/current smoker), heavy alcohol use (>42 units/week), physical activity (inactive/occasional/light/moderate or more/unknown), left ventricular hypertrophy on ECG (yes/no), use of antihypertensive drugs (yes/no) and diabetes mellitus at baseline (yes/no) (modeled as categorical variables). To obtain standardized β-coefficients, they were also repeated with log CRP and log IL-6 modeled as continuous variables in place of CRP and IL-6 quartiles. These analyses were then repeated with both CRP and IL-6 together in the same model, to compare the relative associations of the two with NT-proBNP.

To examine the associations between inflammatory markers and HF risk, participants were divided into quartiles based on distributions of CRP and (separately) for IL-6. Kaplan–Meier curves and the log rank test was used to evaluate differences in HF rates for the four CRP and IL-6 groups. Cox proportional hazard modelling was then used to assess the multivariate-adjusted relative risk of incident HF for CRP and IL-6 quartiles relative to the lowest quartile. Subjects who died without a diagnosis of HF were censored at the time of death, as were those who were alive and free of HF at record review in June 2016. The proportional hazards assumption was examined using time varying covariates, calculating interactions of CRP/IL-6 and a function of survival time and including them in the models.

The proportional hazards assumption was not met for CRP. The assumption of proportionality of hazards was violated at approximately 12 years of follow-up for the CRP quartiles. A sensitivity analysis was conducted, limiting follow-up to 12 years and censoring all cases beyond that point. A similar pattern of risk was seen as in the main analysis, with elevated HF risk greater in the third versus the first quartile than fourth versus first quartile. We therefore elected to report analyses over the entire follow-up time period.

Multivariate analyses were performed, initially age adjusted and then adjusting for various confounders and for the effects of NT-proBNP. Thus three additional analyses to the age adjustment model were performed, adjusting for: age and BMI, age, BMI and conventional risk factors (social class, systolic blood pressure, use of antihypertensive drugs, diabetes mellitus, serum HDL, smoking status, heavy alcohol use (>42 units/week), physical activity, left ventricular hypertrophy on ECG and eGFR) and age, BMI and log NT-proBNP. Log CRP and log IL-6 were also fitted as continuous variables, in place of CRP and IL-6 quartile, respectively, to obtain a p-value for trend.

To examine whether the IL-6/CRP HF relationship differed according to NT-proBNP levels (interaction between NT-proBNP and IL-6/CRP), we performed three additional analyses: first, we examined the age- and BMI-adjusted association between quartiles of CRP/IL-6 and incident HF, stratified by tertiles of NT-proBNP. Second, we examined the association between log NT-proBNP, adjusted for age and BMI, stratified by tertiles of CRP and IL-6. Third, we carried out a formal test for interaction by including an interaction term (CRP quartile*log NT-proBNP/IL-6 quartile*log NT-proBNP) in the age, BMI and log NT-proBNP-adjusted model for the entire sample.

Supplementary analyses were performed, restricting incident cases to those with available echocardiographic-derived information on left ventricular fraction (180 cases), aiming to determine if any differences appeared in the pattern of risk observed for HFrEF versus HFpEF risk. Cox proportional hazard modelling was used to assess the relative risk of incident HFpEF or HFrEF, adjusted for age.

## Results

### Study population

A total of 4252 men (72% of the survivors) attended the examination in 1998–2000. A total of 130 men who had a prior diagnosis of HF (according to self-report or physician diagnosis) were excluded from this analysis; a further 424 men with prior MI were excluded, as were 129 men for whom measurements of both CRP and IL-6 were unavailable. This left 3569 men included in the analysis.

Of these 3569 men, 327 developed incident HF during a median follow-up time of 16.3 years (5.62 cases per 1000 men per year). A total of 476 men sustained a MI (median time at risk 16.0 years, 10.3 cases per 1000 men per year). A total of 1854 men died during follow-up.

Echocardiographic information was available for only 55% (180) of the 327 incident HF cases (while the other patients are likely to have had echocardiograms as part of their clinical diagnostic workup, we lacked information on the results). Of these, 134 had probable HFrEF and 46 has probable HFpEF.

### Baseline characteristics of study participants

Table 1 presents the baseline characteristics of men who did, and did not, develop incident HF. Mean age, BMI, systolic blood pressure, NT-proBNP, CRP and IL-6 were statistically significantly higher in the group developing incident HF; mean eGFR was significantly lower in the incident HF group. Men who developed incident HF were significantly more likely to have had atrial fibrillation at baseline.

**Table 1.** Baseline characteristics of the study population.

Characteristics	Did not develop HF (n = 3242)	Developed HF (n = 327)	p-value
Age (years)	68.4 (5.47)	69.7 (5.40)	<0.0001
**Smoking status**			0.1773
Never smoked (%)	993 (30.7)	88 (26.9)	
Long term ex-smoker (%)	1442 (44.6)	152 (46.5)	
Recent ex-smoker (%)	377 (11.7)	49 (15)	
Current smoker (%)	425 (13.1)	38 (11.6)	
**Physical activity**			0.1176
Inactive (%)	312 (9.62)	30 (9.17)	
Occasional (%)	711 (21.9)	81 (24.8)	
Light (%)	586 (18.1)	69 (21.1)	
Moderate or above (%)	1512 (46.6)	142 (43.4)	
Unknown (%)	121 (3.73)	5 (1.53)	
**Social class**			0.99
Manual occupation (%)	1512 (46.8)	153 (46.8)	
Nonmanual occupation (%)	1722 (53.3)	174 (53.2)	
Heavy alcohol use (%)	88 (2.71)	15 (4.59)	0.054
Taking antihypertensive medication (%)	850 (26.6)	121 (37.5)	<0.0001
Diabetes mellitus (%)	339 (10.5)	45 (13.8)	0.0633
**Clinical characteristics**			
BMI (kg/m^2^)	26.7 (3.56)	27.6 (3.8)	<0.0001
Systolic blood pressure (mmHg)	150 (23.8)	153 (25.1)	0.012
Diastolic blood pressure (mmHg)	85.7 (11.1)	85.9 (10.9)	0.736
**Electrocardiographic diagnoses**			
Atrial fibrillation (%)	91 (2.81)	26 (7.98)	<0.0001
Left ventricular hypertrophy (%)	180 (5.57)	26 (7.98)	0.0757
**Laboratory measurements**			
N-terminal pro-B-type natriuretic peptide (pg/ml)	82.7 (41–155)	169 (70–378)	<0.0001
C-reactive protein (mg/l)	1.66 (0.80–3.33)	1.97 (0.97–3.56)	0.0059
IL-6 (pg/ml)	2.37 (1.53–3.34)	2.69 (1.72–3.66)	0.0006
High-density lipoprotein (mmol/l)	1.33 (0.34)	1.30 (0.35)	0.1154
Estimated glomerular filtration rate (ml/min/1.73 m^2^)	73.0 (12.2)	71.0 (12.7)	0.0058

Values are n (%) or mean (SD). For NT-proBNP, CRP and IL-6 values are geometric mean (interquartile range).

BNP: B-type natriuretic peptide; CRP: C-reactive protein; SD: Standard deviation.

### Relationships between CRP, IL-6 & NT-proBNP

Table 2 shows the unadjusted and adjusted mean (geometric) NT-proBNP by quartiles of CRP and IL-6, respectively. In a multiple regression model incorporating traditional risk factors, log CRP was significantly associated with log NT-proBNP (standardized β-coefficient = 0.115, p < 0.0001). Log IL-6 was also associated with log NT-proBNP, with a larger effect (standardized β-coefficient = 0.146, p < 0.0001).

**Table 2.** Geometric means of NT-pro-B-type natriuretic peptide (pg/ml), unadjusted, adjusted for age and fully adjusted, by quartiles of C-reactive protein and IL-6 and standardized β-coefficients.

CRP	First quartile (<0.81 mg/l)	Second quartile (0.81–1.54 mg/l)	Third quartile (1.55–3.35 mg/l)	Fourth quartile (>3.36 mg/l)	Standardized β-coefficient (standard error)	R-square	p-value for trend
Crude geometric mean NT-proBNP (pg/ml) (interquartile range)	68.2 (36–123)	82.2 (42–147)	91.7 (45–176)	119.4 (56–245)	0.222 (0.019)	0.04	<0.0001
Age-adjusted geometric mean NT-proBNP (pg/ml)	75.5	82.7	89.3	109.8	0.150 (0.018)	0.18	<0.0001
Fully adjusted geometric mean NT-proBNP (pg/ml)	78.4	83.3	87.5	104.3	0.115 (0.018)	0.35	<0.0001
IL-6	First quartile (<1.55 pg/ml)	Second quartile (1.55–2.19 pg/ml)	Third quartile (2.20–3.39 pg/ml)	Fourth quartile (>3.40 pg/ml)			
Crude geometric mean NT-proBNP (pg/ml) (interquartile range)	63.2 (34–115)	77.6 (41–147)	100.6 (50–187)	124.0 (58–266)	0.274 (0.019)	0.06	<0.0001
Age-adjusted geometric mean NT-proBNP (pg/ml)	72.8	78.6	95.3	111.9	0.183 (0.018)	0.19	<0.0001
Fully adjusted geometric mean NT-proBNP (pg/ml)	75.5	82.0	92.7	103.4	0.146 (0.018)	0.33	<0.0001

‘Fully adjusted means’ are adjusted for age, BMI, HDL, eGFR, systolic blood pressure, social class, use of antihypertensive medication, diabetes mellitus, heavy alcohol use, physical activity, left ventricular hypertrophy on ECG, atrial fibrillation on ECG, and smoking status. Standardized β-coefficients, R-square and p-values are given for a regression model incorporating log CRP or log IL-6 in place of CRP or IL-6 quartile, with the standardized β-coefficients representing the increase in log NT-proBNP (as standard deviations) for each 1 standard deviation increase in log CRP or log IL-6.

BNP: B-type natriuretic peptide; CRP: C-reactive protein; eGFR: Estimated glomerular filtration rate; HDL: High-density lipoprotein.

In a multiple regression model including both log CRP and log IL-6 alongside traditional risk factors, the association with log NT-proBNP remained stronger for log IL-6 than log CRP (standardized β-coefficient = 0.058 for log CRP, p = 0.0047, standardized β-coefficient = 0.119 for log IL-6, p < 0.0001).

### Inflammatory markers & risk of incident HF

Kaplan–Meier analysis of risk of incident HF by quartiles of CRP and IL-6 in men without prevalent MI or HF showed that the risk of HF increased with increasing levels of CRP and IL-6 (log rank test both p < 0.0001). Kaplan–Meier graphs are shown in Supplementary Figure 1A & 1B.

**Figure 1.**
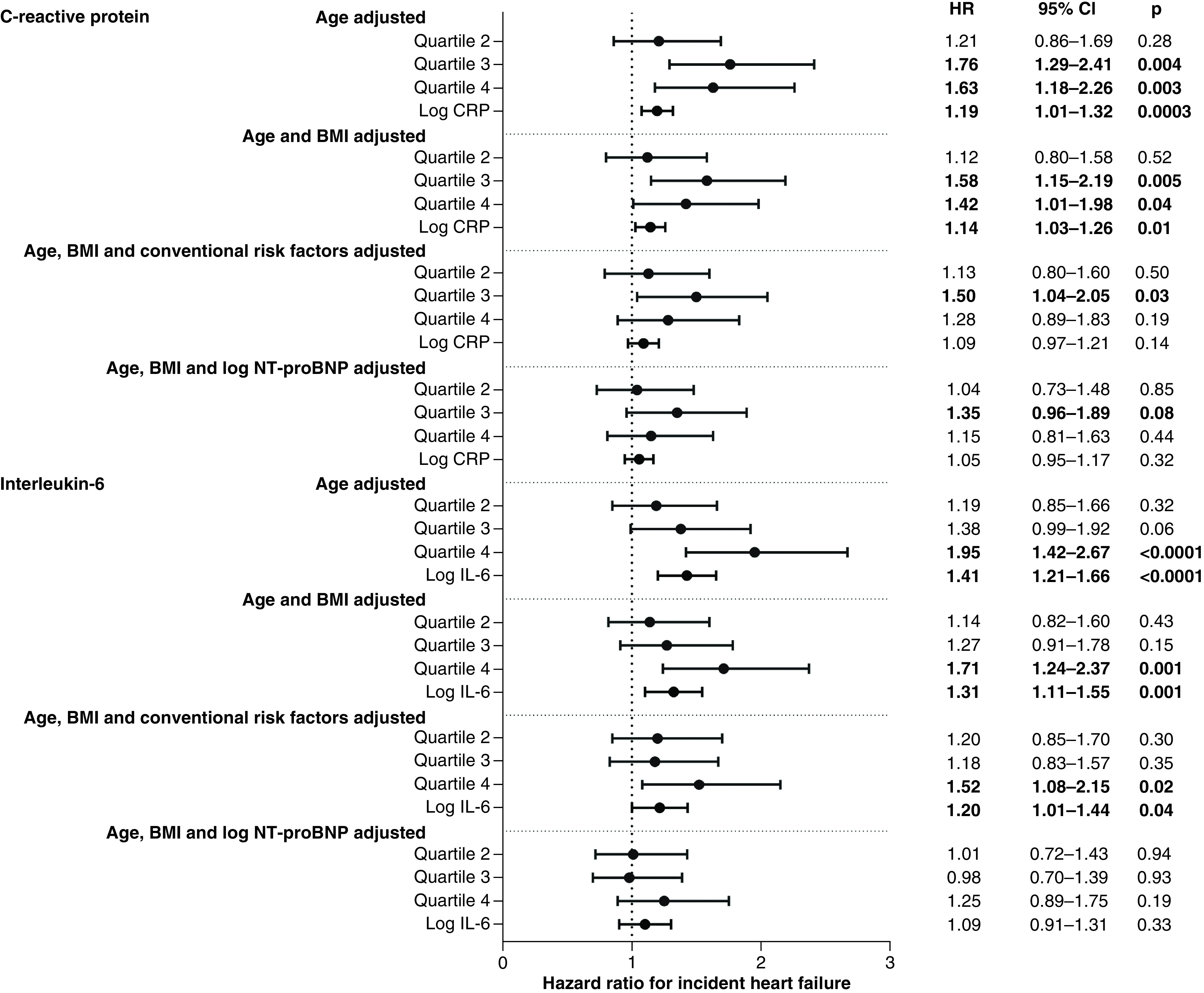
Adjusted relative hazard ratios and 95% CI: for incident heart failure, by quartiles of C-reactive protein and IL-6 and with log C-reactive protein/IL-6 fitted continuously, in men with no prevalent myocardial infarction or heart failure. Adjustment for ‘conventional risk factors’ denotes adjustment for: social class; systolic blood pressure; use of antihypertensive drugs; diabetes mellitus; high-density lipoprotein; smoking status; heavy alcohol use; physical activity; left ventricular hypertrophy on ECG and estimated glomerular filtration rate. For quartiles, HR is relative to first quartile of CRP/IL-6. For log CRP/IL-6, HR is per 1-unit increase in log CRP/IL-6. Hazard ratios at p < 0.05 are bolded.

Figure 1 shows the results of stepwise Cox proportional hazard modelling of incident HF risk for the second, third and fourth quartiles of CRP or IL-6 versus the first quartile of CRP or IL-6. Table 3 shows the incidence rates of HF per 1000 person-years for each quartile.

**Table 3.** Incidence rates of heart failure/1000 person-years, missing values and numbers of men in each C-reactive protein and IL-6 quartile.

CRP (missing values = 25)	First quartile (<0.81 mg/l) n = 888	Second quartile (0.81–1.54 mg/l) n = 886	Third quartile (1.55–3.35 mg/l) n = 884	Fourth quartile (>3.36 mg/l) n = 883
Rate/1000 person-years (327 cases)	4.94	6.25	9.31	8.85

IL-6 (missing values = 30)	First quartile (<1.55 pg/ml) n = 889	Second quartile (1.55–2.19 pg/ml) n = 876	Third quartile (2.20–3.39 pg/ml) n = 886	Fourth quartile (>3.40 pg/ml) n = 888
Rate/1000 person-years (325 cases)	4.91	6.27	7.58	10.8

CRP: C-reactive protein.

There was a positive association between CRP and incident HF after adjustment for age and BMI, but this was attenuated after adjustment for conventional risk factors, though those in the third quartile of CRP showed significantly increased risk. A positive association was seen between IL-6 and incident HF, which remained after adjustment for conventional risk factors, with risk significantly raised in the top quartile. However, adjustment for NT-proBNP abolished the associations between both CRP and IL-6 and incident HF.

### Interactions between CRP/IL-6 & NT-proBNP

Baseline characteristics, rates of incident HF, and distributions among inflammatory marker quartiles by tertile of NT-proBNP are shown in Table 4. In an age- and BMI-adjusted Cox proportional hazard model, HF risk was associated with elevated baseline IL-6 for those participants in the top tertile of NT-proBNP (HR 1.76 for fourth vs first IL-6 quartile, 95% CI: 1.09–2.86, p = 0.022), but not in the middle or bottom tertiles (HR 0.92, 95% CI: 0.51–1.66, p = 0.79 and HR 1.43, 95% CI: 0.59–3.5, p = 0.42, respectively). As seen in the main analyses, adjusting for log NT-proBNP abolished the association between HF risk and IL-6 seen in the top tertile. There were no statistically significant associations between CRP quartile and HF risk in any of the NT-proBNP tertiles.

**Table 4.** Baseline characteristics, incident heart failure rates, and distributions among inflammatory marker quartiles, by tertile of NT-pro-B-type natriuretic peptide.

Characteristics	First tertile of NT-proBNP (<55 pg/ml) n = 1099	Second tertile of NT-proBNP (55–125 pg/ml) n = 1101	Third tertile of NT-proBNP (>126 pg/ml) n = 1101
Age (years)	66 (4.6)	68 (5.3)	71 (5.4)
BMI (kg/m^2^)	27.0 (3.4)	26.8 (3.5)	26.6 (3.8)
Incident HF cases (%)	45^†^ (4)	88^‡^ (8)	174^§^ (16)
HF rate (per 1000 person-years)	2.78	5.98	15.01
First quartile CRP (%)	345 (32)	277 (25)	203 (19)
Second quartile CRP (%)	292 (27)	289 (26)	243 (22)
Third quartile CRP (%)	258 (24)	266 (24)	293 (27)
Fourth quartile CRP (%)	198 (18)	262 (24)	354 (32)
First quartile IL-6 (%)	360 (33)	281 (26)	180 (17)
Second quartile IL-6 (%)	308 (28)	270 (25)	244 (22)
Third quartile IL-6 (%)	234 (21)	292 (27)	301 (28)
Fourth quartile IL-6 (%)	190 (17)	252 (23)	365 (33)

For age and BMI, values are mean (SD). For HF cases and CRP/IL-6 quartiles, values are n (%).

† Five cases of probable HFpEF, 18 cases of probable HFrEF, 22 cases of HF unknown subtype.

‡ 18 cases of probable HFpEF, 39 cases of probable HFrEF, 23 cases of HF unknown subtype.

§ 23 cases of probable HFpEF, 67 cases of probable HFrEF, 84 cases of HF unknown subtype.

BNP: B-type natriuretic peptide; CRP: C-reactive protein; HF: Heart failure; HFrEF: HF with reduced ejection fraction.

In subgroup analyses restricted to each tertile of CRP and IL-6, log NT-proBNP was highly statistically significantly associated with HF risk in all cases (p < 0.0001).

A formal test for interaction showed no significant interaction between IL-6/CRP and NT-proBNP with incident HF.

### Supplementary analysis of incident HFrEF & HFpEF

We also conducted a supplementary analysis examining the associations between inflammatory markers and specific HF types, restricting incident cases to those with information on LVEF (n = 180 cases).

In age-adjusted Cox proportional hazard models, there were trends toward increased incident HFpEF risk at higher quartiles of CRP (HR 2.28 for third vs first quartile; 95% CI: 0.93–5.61) and IL-6 (HR 1.54 for first vs fourth quartile; 95% CI: 0.67–3.53), although these did not reach statistical significance possibly due to the small number of HFpEF cases (n = 46).

There were similar trends toward increased HFrEF risk (n = 134 cases) at higher quartiles of CRP (HR 1.55 for third vs first CRP quartiles; 95% CI: 0.98–2.48) and IL-6 (fourth versus first IL-6 quartiles (HR 1.42; 95% CI: 0.86–2.33); again, these did not reach statistical significance.

## Discussion

In this study, both elevated CRP and elevated IL-6 at baseline were associated with an increased risk of incident HF in older men, over a median follow-up time of 16.3 years, even after accounting for traditional risk factors for HF. However, this association disappeared when adjusting only for age, BMI and NT-proBNP, and, in a subgroup analysis, associations between IL-6 and HF risk were seen only in participants with NT-proBNP levels in the top third of the cohort, but this too was abolished after additional adjustment for NT-proBNP. This suggests that, in this population, NT-proBNP levels, a marker of BNP production, and in turn of NP activity and myocardial stretch, seem to account for much of the association between inflammation and HF risk.

Both CRP and IL-6 levels were associated with raised NT-proBNP levels, but the strength of this association was greater for IL-6 than for CRP. The trend toward increased HF risk with elevated biomarker concentration was also clearer for IL-6 than CRP. IL-6 is an upstream inflammatory marker which, as well as increasing hepatic CRP production, also has wider effects such as coronary plaque initiation and destabilization and microvascular flow dysfunction [19]. It might therefore be a more proximal and specific mediator of chronic inflammatory risk in HF, and may also relate more closely to the activity of the NP system.

The incidence of HF in this study (5.6 cases/1000 person-years) is lower than has been reported in other cohorts (7.1 cases/1000 person-years in men and women aged 45–64 years at baseline in the ARIC study [26] and 15.1 cases/1000 person-years in men and women aged 70–79 years at baseline in the HABC study [8]). These differences may relate to our exclusion of men with prevalent MI at baseline, who are at higher risk of HF.

In asymptomatic individuals, IL-6 levels has shown to be correlated with the degree of left ventricular dysfunction as measured by cardiac magnetic resonance imaging, even after adjusting for demographics, cardiovascular risk factors and markers of subclinical atherosclerosis [37]. Production of proBNP, the precursor molecule of BNP and NT-proBNP, is thought to be predominantly dependent on myocardial wall stretch [38]. The observed relationship in our study might therefore be explained by inflammation leading to subclinical ventricular dysfunction and resultant strain, in turn leading to BNP and NT-proBNP release as a compensatory response.

Further evidence suggests close associations between inflammation and NP levels. In asymptomatic people with hypertension, plasma and coronary sinus BNP levels (directly-measured, as opposed to NT-proBNP) correlate with blood markers of collagen turnover and inflammatory cytokines and with echocardiographic features of cardiac remodeling [39]. Administration of lipopolysaccharides – a potent pro-inflammatory stimulus – to healthy volunteers produces an increase in plasma NT-proBNP [40,41]. Exposing cultured myocytes to pro-inflammatory cytokines (including IL-6) increases ANP and BNP gene expression [42] and BNP synthesis [43]. In elderly people, elevated inflammatory markers are associated with elevated NT-proBNP and an elevated NT-proBNP/BNP ratio [44]. NT-proBNP levels also correlate with the severity of periodontitis, an infective/inflammatory condition itself associated with increased cardiovascular risk [45]. IL-6 receptor blockade in rheumatoid arthritis lowers both disease severity and NT-proBNP levels [46]. Finally, a recent study demonstrated that higher circulating IL-6 levels (although not CRP) were associated with higher NT-proBNP levels in a large community-dwelling cohort; in hospitalized patients, acute respiratory tract infections and sepsis were associated with higher plasma BNP levels, even in those without HF; positive associations were also seen between white cell count, CRP and BNP levels [41].

BNP itself appears to exert an immunomodulatory effect. *In vitro*, adding BNP stimulates macrophage production of pro-inflammatory substances including reactive oxygen species, nitrates and leukotriene B_4_, although it also stimulates production and release of IL-10 (an anti-inflammatory cytokine) and prostaglandin E_2_ (capable of pro- or anti-inflammatory effects depending on context) [47]. NPs appear to have some protective, anti-inflammatory effects: in animal models, atrial NP attenuates inflammatory-related cardiac remodeling [48], and reduces brain injury in sepsis [49]; and C-type NP administration diminishes severity of myocarditis [50]. Overall, NP activity might be an adaptive response in pro-inflammatory states and may reduce the deleterious effects of inflammation.

In supplementary analyses, there were nonsignificant trends toward increased risk of both HFrEF and HFpEF with elevated baseline CRP and IL-6. Results from other cohort studies have been mixed as to the associations between inflammatory markers and subtypes of HF: two analyses of the Health ABC cohort reported an association between inflammation and incident HFpEF, but not HFrEF [8,51], whereas a more recent analysis of pooled data from four large cohort studies found associations between inflammation and incident HFrEF, but not HFpEF [29]. In our group, the trend toward increased risk appeared greatest for HFpEF in participants with elevated baseline CRP, which may support the findings of the Health ABC studies, but both subtypes of HF did show a trend toward increased risk with elevated inflammation, and, of course, in the small subgroup analyses with a small number of events we were unable to demonstrate statistical significance. HFpEF especially appears to be a highly heterogenous disorder with multiple different phenotypes and further characterization of those may help to refine understanding of the role inflammation plays in those conditions [52].

Newer biomarkers, such as mid regional pro-adrenomedullin (MR-proADM) and sST2 hold significant potential for prediction and diagnosis of HF, and may augment the use of NP measurement [7,18,53,54]. MR-proADM and sST2 production are influenced, in part, by the hemodynamics of a fluid-overloaded state in HF, including endothelial shear stress [55], myocyte strain [56,57] and alveolar strain in pulmonary edema [58]. Production of both MR-proADM and sST2 also seem, like NPs, to be influenced by pro-inflammatory states [59–62]. Thus, these biomarkers and their related pathways are likely also involved in the complex relationship between inflammation and HF. Our work could be extended by accounting for the role of these newer markers in the relationship between NPs, inflammation and HF.

### Study limitations

This is a large study, reporting findings from multiple detailed assessments, with long follow-up times. However, there are limitations to this work. We had no baseline echocardiographic data on participants, meaning that the associations reported here between inflammation and HF risk might be due to the inclusion of individuals with asymptomatic or undiagnosed left ventricular dysfunction that may have been apparent on echocardiogram. The current findings are based on physician-diagnosed HF, which is likely to underestimate the true incidence of HF in the study population. However, the other associations with HF risk in this report and in our previous report on obesity, NT-proBNP and lung function and HF [25,63,64] generally accord with prior data and therefore suggest external validity of our findings.

Follow-up echocardiographic data was also sparse, making it difficult to confidently classify participants with HF as having HFrEF or HFpEF, and as a result, a significant proportion of individuals with HF could not be assigned to one of these two subgroups. The study population was entirely male, mostly of white origin, and free of prior MI; our findings may not be generalizable to women, other ethnic groups, and those with prior ischemic heart disease.

## Conclusion

In this study of older men, inflammation, as measured by circulating CRP and IL-6 levels, is associated with an increased risk of incident HF. However, this association was markedly attenuated by the addition of NT-proBNP to risk models. NT-proBNP levels were associated with increased CRP and, more strongly, with increased IL-6 levels at baseline and the increased risk of HF associated with elevated IL-6 was only evident in those with high levels of NT-proBNP. In older men, the activity of the NP system appears, at least in part, linked to inflammatory activity, and the elevated risk of HF seen in individuals with higher circulating inflammatory markers seems to be associated with NT-proBNP levels.

## Future perspective

The mechanism of links between NP activity and inflammation are likely to be further elucidated, and we expect pathophysiological understanding of how these relate to the development of subsequent HF to increase. The differentiation between HF with preserved ejection fraction and reduced ejection fraction is apparent in clinical practice and is likely to be made more explicit in future research; differential associations between inflammation, NP activity and HFrEF/HFpEF are likely to emerge to suggest differing pathophysiology. From a therapeutic standpoint, efforts to use anti-inflammatory therapy for HF have largely been disappointing, though therapies targeting NP activity (e.g., sacubitril) have been more successful, and are currently in use; further therapeutic options, again likely targeted at either HFpEF or HFrEF, are likely to be developed.

**Summary points**

- Natriuretic peptide (NP) activity is very strongly associated with heart failure (HF).
- Associations between pro-inflammatory biomarkers and incident HF have been described prior, but few of these studies accounted for NP activity.
- Laboratory and clinical studies suggest inflammatory and NP activity are linked to one another.
- We found that, in a large cohort of older men, C-reactive protein and interleukin-6 levels were associated with NT-pro-B-type natriuretic peptide levels at baseline, even after adjusting for likely confounders.
- During follow-up, elevated C-reactive protein and interleukin-6 levels were associated with increased incident HF risk in models adjusting for age and BMI.
- However, this risk disappeared on addition of baseline NT-pro-B-type natriuretic peptide to the model.
- NP activity is linked to pro-inflammatory biomarker activity.
- These links may be important for determining the mediators of future HF risk, and the two systems may share a common driving force.
