# Supplementary figures and images for: Inflammatory markers and incident heart failure in older men: the role of NT-proBNP
Source: Biomark Med. 2021 Mar 12;15(6):413–25. doi: 10.2217/bmm-2020-0669 (PMC8559131; doi:10.2217/bmm-2020-0669)

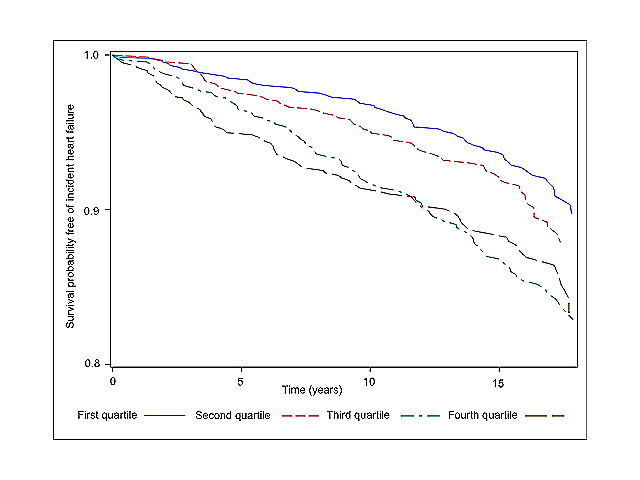

Supplement: Supplementary file 1 [file bmm-15-413-s1.png]

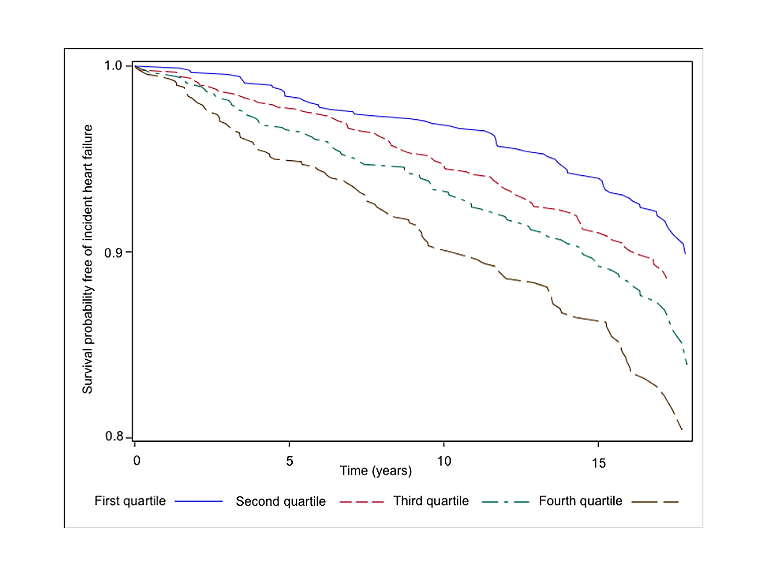

Supplement: Supplementary file 2 [file bmm-15-413-s2.png]
